# Intralabyrinthine schwannomas: a two-case series and literature review with a focus on hearing rehabilitation

**DOI:** 10.1007/s00405-023-07823-2

**Published:** 2023-01-17

**Authors:** Luigi Curatoli, Vito Pontillo, Nicola Quaranta

**Affiliations:** Translational Biomedicine and Neurosciences Department, University of Bari, Bari, Italy

**Keywords:** Intralabyrinthine schwannoma, Cochlear implantation, Hearing rehabilitation, Inner ear

## Abstract

**Purpose:**

Intralabyrinthine schwannomas (ILSs) are an uncommon finding. Diagnosis is challenging and no gold standard treatment exists yet. In this article, we present a two-cases series and review the latest available literature to assess the best diagnostic and therapeutic scheme.

**Methods:**

We reviewed the latest available literature assessing most frequent and relevant sets of symptoms, clinical features of the disease, diagnostic tests and imaging, possible treatments and after-surgery hearing rehabilitation techniques. We then compared literature data to our own series ones.

**Results:**

ILSs clinical presentation and development may overlap with other, more common otological conditions. Full audiometric battery test, electrophysiological study of VEMPS and MRI with contrast enhancement all appear to be critical to correctly diagnose these tumors. Several treatments exist: radiological follow-up, radiation therapy, full or partial surgical excision. Hearing rehabilitation is mostly accomplished through simultaneous cochlear implantation.

**Conclusions:**

Our case-series data matches the available literature. ILSs are a rare type of vestibular schwannomas. Diagnosis in challenging and delayed in time as all the diagnostic tests, yet sensitive, are not specific for ILSs. The most suitable treatment seems to be surgical excision of these tumors followed by simultaneous cochlear implantation to restore hearing.

## Introduction

Intralabyrinthine schwannomas (ILSs) are a specific group of 8th nerve schwannomas that are located within the labyrinth. Their low prevalence (9% of all vestibular schwannomas—VS [1]), together with their small size and unusual location, make them a real challenge for both radiologists and otolaryngologists. The present paper reports two cases of ILS and a review of the latest available literature on the diagnosis and treatment options, with a special focus on hearing rehabilitation.

## Cases

### Case 1

**Fig. 1:**
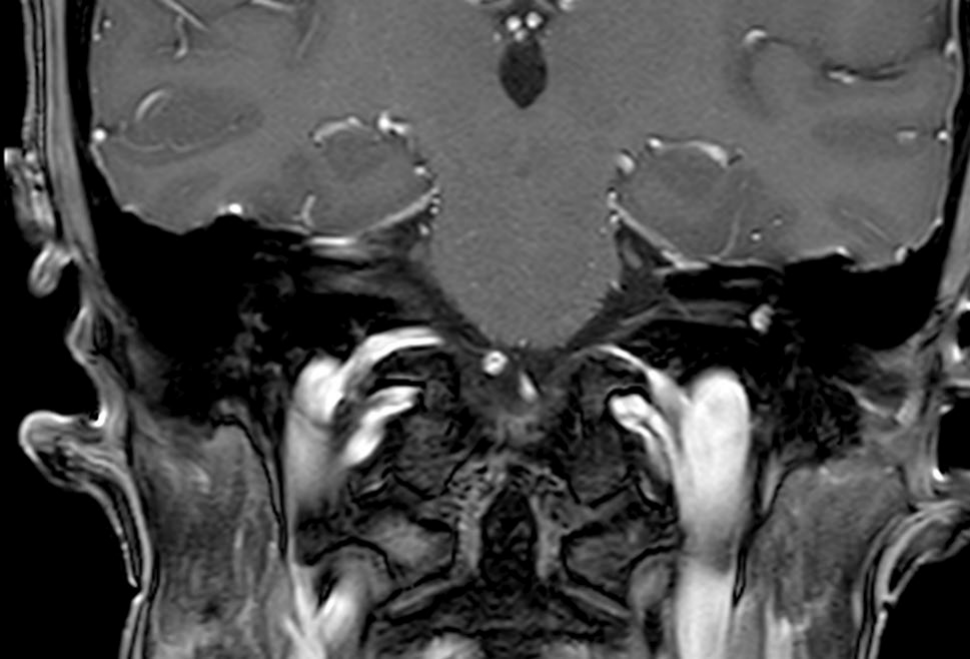
Coronal T1 gadolinium enhanced MR showing an enhancing lesion in the left vestibule

A 60-year-old female patient came to our attention in late 2021 complaining of recurrent vertigo and left-sided tinnitus since November 2017, and reporting an episode of left sudden sensorineural hearing loss (SSNHL) in March 2018. She had a positive clinical history for patent foramen ovale surgery and an autoimmune thyroiditis. The patient had undergone two Magnetic Resonance Imaging (MRI) scans in 2018 and in 2019, both reported as negative for cerebellopontine angle (CPA), 8th cranial nerve and inner ear pathology. In late 2021 she underwent a third MRI scan that showed a 5 × 3 mm contrast enhanced lesion filling the vestibule and the ampullary end of the posterior semicircular canal (Fig. 1). Audiometry showed normal hearing thresholds in the right ear (10 dB HL Pure Tone Average-PTA for the frequencies between 500 and 3000 Hz and 100% Speech Discrimination Score-SDS) and profound sensorineural hearing loss (SNHL) in the left ear (> 120 dB HL PTA and 0% SDS). Facial nerve function was normal, bedside vestibular evaluation showed a normal vestibulo-ocular reflex (VOR) on both sides. The patient underwent a trans-labyrinthine removal of the lesion (histologically reported as a type A schwannoma) with simultaneous cochlear implantation (CI) (Cochlear Nucleus CI612) through a posterior tympanotomy approach. Intraoperative electrophysiological measures showed normal values of impedance and NRT (neural response telemetry) over all electrodes and a Trans Impedance Matrix (TIM) not suggestive of tip fold-over. Six months after CI activation PTA was 29 dB HL and SDS 100% in the implanted ear with contralateral masking; patient reported no further episodes of vertigo and a slightly persistent tinnitus.

### Case 2

**Fig. 2:**
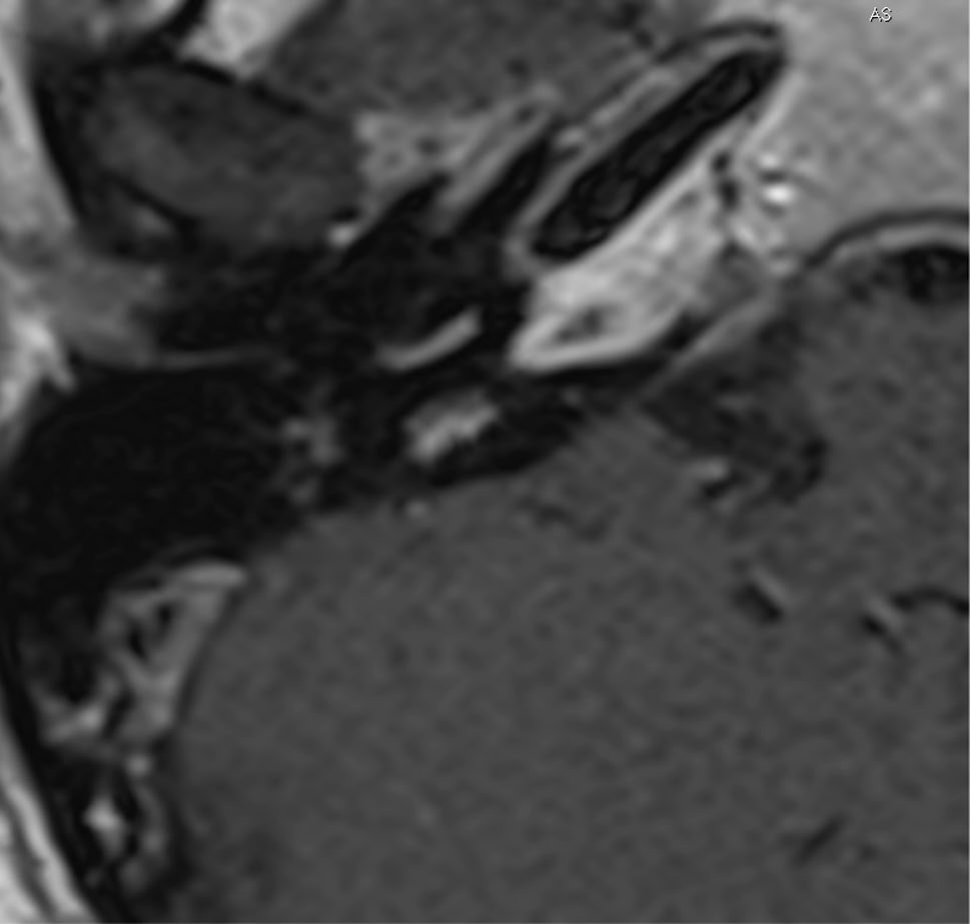
Axial T1 gadolinium enhanced MR showing an enhancing lesion in the right basal turn of the cochlea

**Fig. 3:**
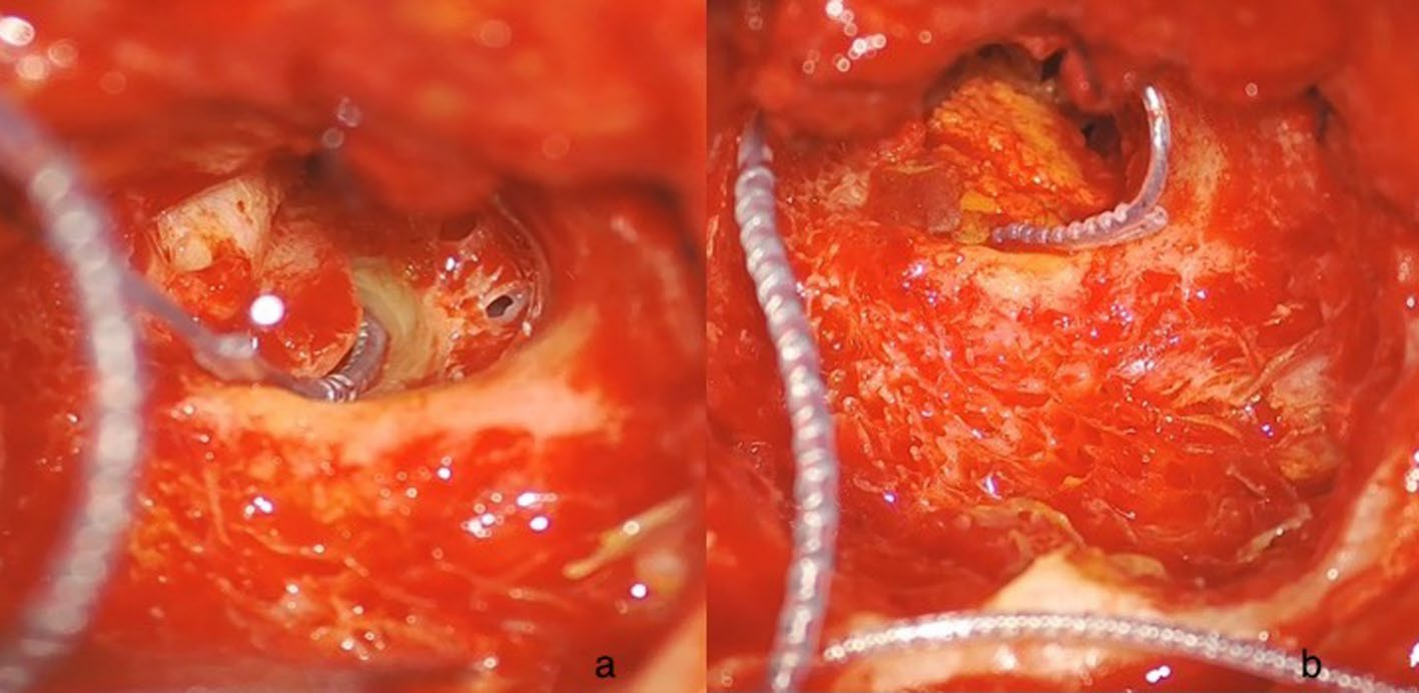
CI after the tumor excision, before (**a**) and after (**b**) the reconstruction of the lateral wall of the cochlea with bone pâté

**Fig. 4:**
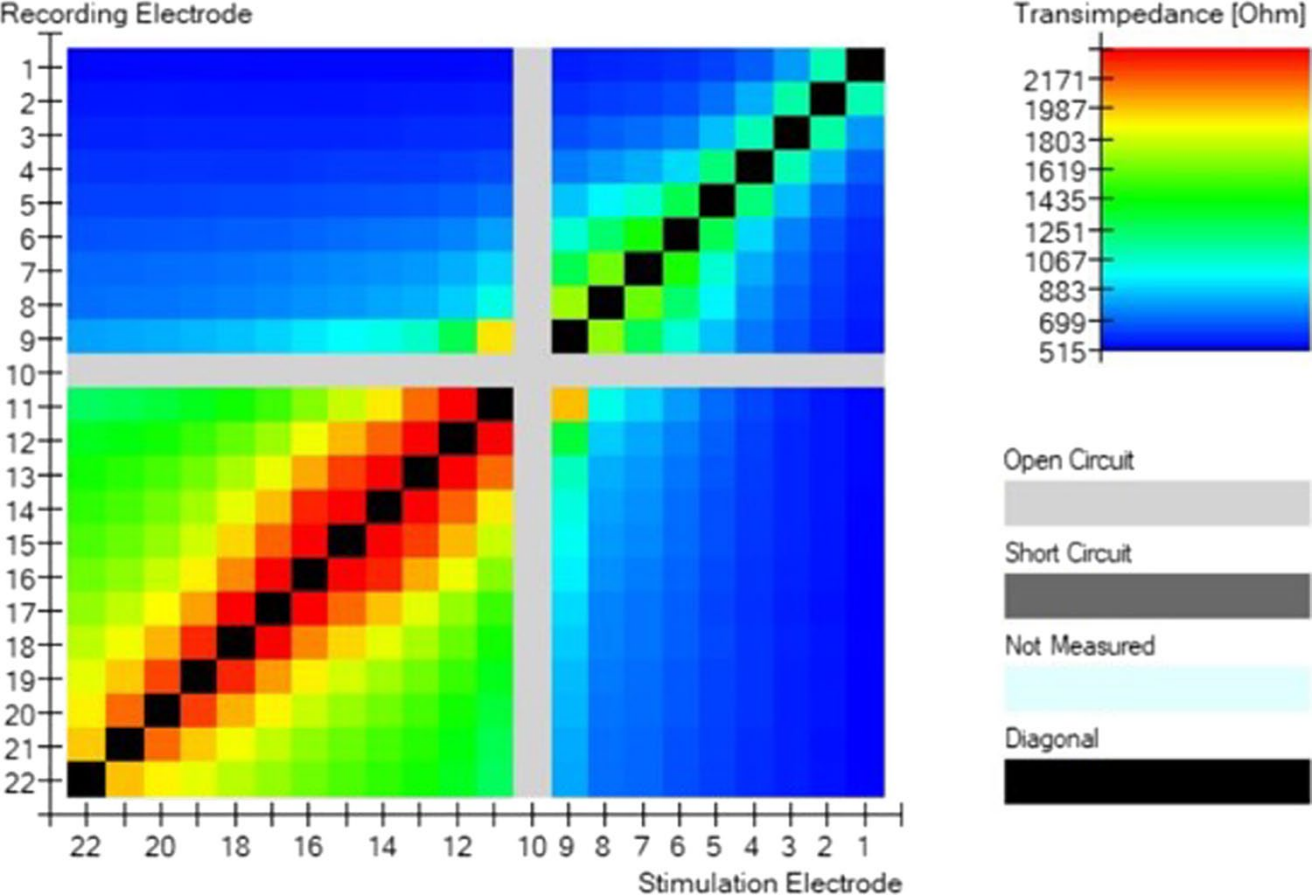
Intraoperative TIM monitoring of CI of patient report in case 2

**Fig. 5:**
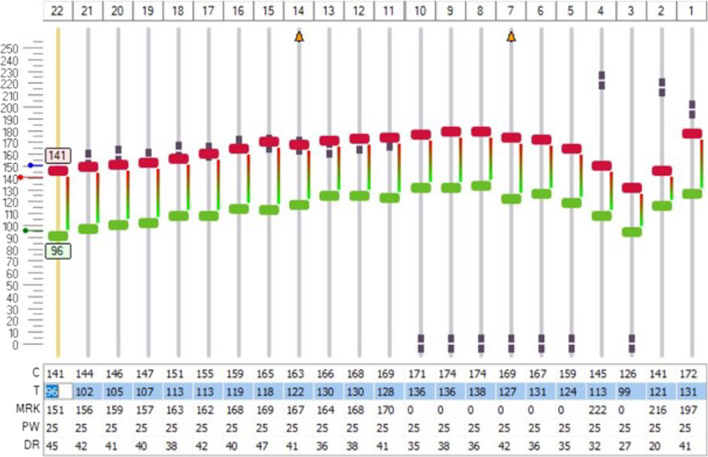
Technical map of CI function after activation of patient reported in case 2

A 57-year-old male patient came to our attention at the beginning of 2022 complaining of right-sided progressive hearing loss and continuous tinnitus since early 2021. He had undergone two different MRI scans, both reported as negative for CPA and 8th cranial nerve disease. MR images re-analysis showed an intracochlear ILS, measuring 7 × 1 mm (Fig. 2). Audiometry showed normal hearing thresholds in the left ear (17.5 dB HL PTA and 100% SDS) and profound SNHL in the right ear (90 dB HL PTA and 20% SDS). Facial nerve function was normal. The patient underwent subtotal petrosectomy and blind sac closure of the external auditory canal. The lateral wall of the basal turn of the cochlear was drilled up to the first genu showing a soft tissue lesion located in the scala tympani. After the schwannoma removal, histologically Antoni type A, a CI Nucleus CI612 was inserted and the lateral wall of the cochlea was reconstructed with bone pâté and temporalis muscle (Fig. 3). Intraoperative measures showed normal values of impedance, an absent NRT on electrodes 3, and 5 to 10, and a normal TIM except for an open circuit on electrode 10 (Fig. 4). All the electrodes were activated (Fig. 5) and 6 months after activation PTA was 35 dB HL and SDS 40% on the implanted side; the patient reported complete resolution of tinnitus.

## Literature review

### Definition, site of origin and classification of ILS.

Vestibular schwannoma (VS) is a common benign tumor, accounting for up to 8% of intracranial neoplasm [2]. VS are usually unilateral in sporadic disease and bilateral in type-two neurofibromatosis [3]. Its site of origin is still debated even if there is now general agreement that it is lateral to the *Obersteiner-Redlich* zone [4, 5], where the glial cover of the nerve is replaced by Schwann cells. ILSs are a rare type of VS, with an estimated prevalence of 0.9% in autoptic studies [6]. In 2004, Kennedy et al. proposed a classification system for ILS based on the precise point of localization of the tumor inside the labyrinth [7], describing seven different types: intravestibular, intracochlear, intravestibulocochlear, transmodiolar, transmacular, transotic and tympanolabyrinthine.

### Clinical presentation

Patients suffering from ILS may present with common symptoms, such as hearing loss (HL), vertigo/dizziness and tinnitus [8]. HL is usually sensorineural [9, 10], but transmissive and mixed HL have been also reported due to the dampening effect on the stapes footplate [7, 11, 12]. Fluctuating HL has also been reported [13]. Therefore, particular attention is required to patients with a previous diagnosis of Ménière disease (MD), as the symptomatology set may be similar and easily confused [13]. Jerin et al. recently described similar characteristics of HL in ILS and MD, differing only in the severity of HL, being worse in case of ILS [14]. Facial nerve palsy may occur, suggesting a more severe and advanced stage of disease [8].

### Diagnosis

The diagnosis of ILS is often delayed in time, due to non-specific symptoms, radiological misdiagnosis, low prevalence, and low growing rate [8]. A complete assessment needs a careful collection of clinical history, a full audiometric battery testing and electrophysiological studies, including VEMPS; while VEMPs are absent or reduced in case of VS [15] they can be normal or even enhanced in case of ILS [8, 13, 16, 17]. The diagnostic gold standard is MRI, showing an hyperintense signal in contrast-enhanced T1-weighted (CE-T1WI) and FLAIR sequences and a hypointense signal in 3D T2-weighted images (3D T2WI) [13, 18–22]. In particular, as recently suggested by Karol et al., contrast may not be essential [23], since non-enhanced T2WI sequences alone may detect ILS with up to 100% sensitivity [24]. The main differential diagnosis must take into account labyrinthitis, as it usually appears as hyperintense in CE-T1WI sequences and isointense in T2WI sequences [17, 20, 22].

### Treatment and hearing rehabilitation

Due to the rarity of these tumors only few cases have been reported so far, hence the lack of gold standard treatment nor guidelines, forcing the otolaryngologist to a case-by-case evaluation of the best therapeutic option. Kennedy et al. suggested that radiologic follow-up with repeated MRI is the best strategy for small ILS causing a non-invalidating symptomatology, given their slow growing rate [7, 20]; nevertheless, this conservative approach may drive to the progressive extension into the IAC with a subsequent increase in surgical morbidity [7, 25]. Pharmacological labyrinthectomy with aminoglycosides has been proposed for patients under radiological surveillance who develop severe vestibular symptoms [10, 19]. Radiation therapy (RT) may be an option in elderly and non-surgery-fit patients, as it may complicate hearing rehabilitation via CI [26, 27].

Surgery represents the best option in case of small tumors that may be completely excised with limited surgical morbidity. Microscopic, endoscopic [28, 29] as well as combined approaches have been described [20, 30]. The surgical approach depends on the location and size of the tumor; in fact, in case of localization in the posterior labyrinth a classical translabyrinthine approach is mandatory, while localization in the cochlea requires the drilling of the cochlea itself. Other techniques have been described, depending on the size and location of the tumor, such as the extended round window approach, the partial or subtotal cochleostomy and the “pull-through-technique” [31].

In terms of hearing rehabilitation, the translabyrinthine approach allows the intracochlear application of a CI through a posterior tympanotomy approach, while drilling the cochlea may prevent the application of a CI, if the modiolus and spiral lamina are not carefully preserved [32, 33]; Ma et al. have argued that a subtotal excision of the tumor may be acceptable if the purpose is sparing these structures [30].

Although a case of intracochlear schwannoma excision with hearing preservation was reported [31], cochlear implantation represents the best option for hearing rehabilitation. It can be placed at the time of tumor removal or during a second-stage surgery; in the latter case, a dummy electrode should be inserted in the cochlea to prevent its ossification [31, 32, 34]. According to the data available in the literature, CI has been successfully carried out in patients affected by different types of ILS (intravestibular, intracochlear, intravestibulocochlear, transmodiolar, transmacular) [25, 26, 32–38]; there is no report of CI after excision of transotic or tympanolabyrinthine tumor.

So far, CI appears to be a feasible hearing rehabilitation strategy as long as the modiolus and the spiral lamina are preserved, despite the extent of the tumor [32, 33].

In case of cochlear drilling, after CI insertion the lateral cochlear wall should be reconstructed with temporalis muscle fascia, autologous cartilage, perichondrium or bone pâté [26].

Auditory outcomes of CI after ILS surgery have been reported in case series and reports [25, 26, 32–38], providing extremely variable postoperative speech discrimination scores, ranging from 0% up to 100%.

Table 1 summarizes the SDSs of the different series according to the location of the lesion and the extent of the removal. The reported results suggest that ILS should be always removed, when possible, especially in cases with no cochlear involvement. In the unfortunate case, where the cochlea is affected, tumor excision should spare the modiolar wall. In case of transmodiolar tumors, a conservative approach should be preferred, especially for small lesions. In particular, the insertion of a CI without tumor removal [25, 31, 38] reduces the risk of surgical cochlear damage, but is associated with worse functional results (less than 30% on test) [25] and with a more challenging MRI follow-up, due to the magnetic interferences of the electrode [38].

**Table 1:** Average speech discrimination value based on ILS type and removal reported in table

	Tumor removal	Total of patients reported	Type of ILS^d^	Average SDS after CI	Range of SDS
Literature data^a^	Yes	24	IC	51.88%	0 – 100%
		3	IV	53.33%	50 – 60%
		4	IVC	40%	5 – 80%
		–	TM	–	–
		1	TMA	Only case reported: 70%	–
	No	3	IC	32.67%	21 – 50%
		1	IV	One case reported: 32%	–
		1	IVC	One case reported: 70%	–
		7	TM	40%	0 – 88%
		–	TMA	–	–
	Partial	1	TM	One case reported: 15%	–
		3	TM + IV	43.33%	0 – 65%
		1	TM + CPA	One case reported: 85%	–
Present series	Yes	1	IC	Case no. 2: 40%	–
		1	IV	Case no. 1: 100%	–

*CI* cochelar implantation, *CPA* cerebello-pontine angle, *IC* intracochlear ILS, *ILS* intralabyrinthine schwannoma, *IV* intravestibular ILS, *IVC* intravestibulocochlear ILS, *TM* transmodiolar ILS, *TMA* transmacular ILS, *SDS* speech discrimination score

^a^[10, 25, 31, 32, 36, 38]

## Discussion

In this series we presented two cases of ILS, an intravestibular and an intracochlear subtype. The clinical history of the two patients was similar, as they both developed progressive non-specific audiological symptoms, and experienced a veritable diagnostic delay, due to several MRI scans reported as negative for inner-ear pathologies (counting an average delay of 30 months). Both the patients were finally treated by surgical removal with simultaneous CI insertion, given the small size of the tumors, the degree of HL and the absence of vestibular symptoms. In case 1 the IV localization of the tumor did not represent an issue for CI, as there was no risk of direct damage of the cochlea, while in case 2, the removal of the IC schwannoma required the demolition of the lateral wall of the cochlea and its immediate reconstruction with bone pâté and temporalis muscle. Our results in terms of hearing rehabilitation match the ones reported in previous literature as regards to the IC schwannoma (SDS 40% vs. 51,88% of the literature, range 0–100%), and are even better in the IV case (SDS 100% vs. 53,33% of the literature, range 50–60%).

## Conclusion

ILS is a rare type of 8th nerve schwannomas that may cause severe distress for patients: sudden SNHL, vertigo and tinnitus are very frequent and may be strongly debilitating. Delayed diagnosis appears to be an actual issue, even if modern MRI technologies can early detect this type of lesion. Surgical excision with simultaneous or secondary CI seems to be a valuable therapeutic option. The two cases we present in this paper generally reflect the main features of this pathology found in the literature so far. Further studies are although necessary to establish standard treatment protocols to assure patients the best outcome possible.

## Data Availability

The data supporting the findings of this paper are available and can be provided if requested.
